# The Parenchyma of Secondary Xylem and Its Critical Role in Tree Defense against Fungal Decay in Relation to the CODIT Model

**DOI:** 10.3389/fpls.2016.01665

**Published:** 2016-11-09

**Authors:** Hugh Morris, Craig Brodersen, Francis W. M. R. Schwarze, Steven Jansen

**Affiliations:** ^1^Institute of Systematic Botany and Ecology, Ulm UniversityUlm, Germany; ^2^School of Forestry and Environmental Studies, Yale University, New HavenCT, USA; ^3^Laboratory for Applied Wood Materials, Empa-Swiss Federal Laboratories for Materials Testing and ResearchSt. Gallen, Switzerland

**Keywords:** ray parenchyma, axial parenchyma, CODIT, reaction zone, secondary xylem, fungi, barrier zone

## Abstract

This review examines the roles that ray and axial parenchyma (RAP) plays against fungal pathogens in the secondary xylem of wood within the context of the CODIT model (Compartmentalization of Decay in Trees), a defense concept first conceived in the early 1970s by Alex Shigo. This model, simplistic in its design, shows how a large woody perennial is highly compartmented. Anatomical divisions in place at the time of infection or damage, (physical defense) alongside the ‘active’ response by the RAP during and after wounding work together in forming boundaries that function to restrict air or decay spread. The living parenchyma cells play a critical role in all of the four walls (differing anatomical constructs) that the model comprises. To understand how living cells in each of the walls of CODIT cooperate, we must have a clear vision of their complex interconnectivity from a three-dimensional perspective, along with knowledge of the huge variation in ray parenchyma (RP) and axial parenchyma (AP) abundance and patterns. Crucial patterns for defense encompass the symplastic continuum between both RP and AP and the dead tissues, with the latter including the vessel elements, libriform fibers, and imperforate tracheary elements (i.e., vasicentric and vascular tracheids). Also, the heartwood, a chemically altered antimicrobial non-living substance that forms the core of many trees, provides an integral part of the defense system. In the heartwood, dead RAP can play an important role in defense, depending on the genetic constitution of the species. Considering the array of functions that RAP are associated with, from capacitance, through to storage, and long-distance water transport, deciding how their role in defense fits into this suite of functions is a challenge for plant scientists, and likely depends on a range of factors. Here, we explore the important role of RAP in defense against fungal pathogens and the trade-offs involved from a viewpoint for structure-function relations, while also examining how fungi can breach the defense system using an array of enzymes in conjunction with the physically intrusive hyphae.

## Introduction

A knowledge of the anatomy and function of the secondary xylem of trees is critically important to our understanding of defense against pathogens, defined here as disease/decay causing organisms (Carlquist, 2001; Evert and Eichhorn, 2006); to a greater extent against pathogenic fungi, the most studied group (Pearce, 1996; Schwarze et al., 2000c; Schmidt, 2006), but also against bacterial infections, where both types are major factors in tree decline. Also, combining wood structure-function with tree defense strategies can help further our understanding of whole-plant function, which is especially relevant in light of a changing climate (Anderson et al., 2004; Woods et al., 2005; Aitken et al., 2008; Sturrock et al., 2011). Drought, in particular, caused by altered patterns in both the severity and return frequency, and an overall reduction in precipitation, is strongly linked to xylem hydraulic failure (Anderegg et al., 2012; Choat et al., 2012; Nardini et al., 2013; Sevanto et al., 2014), and is a major precursor to disease out-breaks (Bréda et al., 2006; Sturrock et al., 2011). Indeed, disease outbreaks can be indirectly caused by beetles attracted to the chemicals given off by drought-stressed trees (Anderegg et al., 2015), which carry the disease causing agent (Addison et al., 2014; Reed et al., 2014). An example is the association between the mountain pine beetle (*Dendroctonus ponderosae*) and the blue-stain fungus *Grosmannia clavigera* causing the decline of lodgepole pines in North America, where the fungus attacks the living cell network, gradually spreading throughout the sapwood and causing the death of the tree through starvation (Kirisits, 2007). However, disease can be caused directly by pathogens on trees that are stressed, which may turn latent fungi pathogenic (Griffith and Boddy, 1990; Desprez-Loustau et al., 2006; Parfitt et al., 2010), or on otherwise healthy trees where conditions favor the pathogen, such as is the case with the opportunistic fungus *Armillaria* (Kile et al., 1991).

**FIGURE 1:**
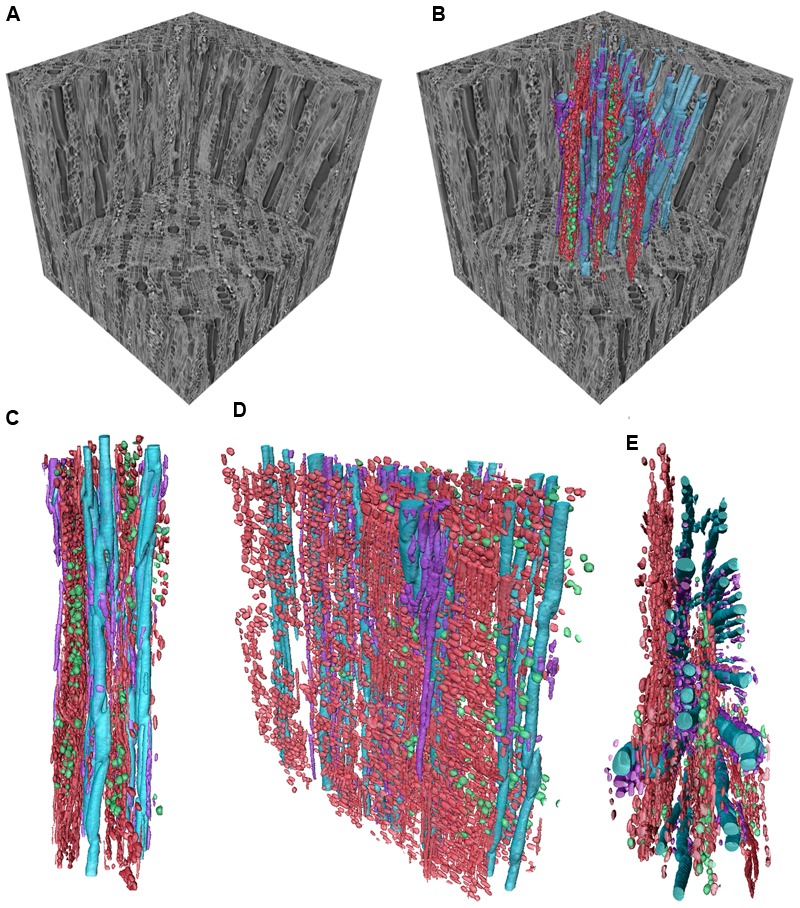
**Three dimensional volume renderings of a *Ziziphus obtusifolia* stem sample presented as an example of the CODIT framework.** Sub-volumes of the larger sample can be visualized in isolation **(A)** or with various tissue types segmented into separate volumes **(B).** In *Ziziphus*, the rays (red) are arranged in a planar orientation, radiating out from the stem center, and create discrete sectors between vessel rows, as visualized from different perspectives, tangential in **(C)**, oblique radial in **(D)**, and transverse in **(E).** The rays form the majority of wall 3 in the CODIT framework. The AP (purple) surround the vessels (blue). Calcium oxalate crystals can be found distributed throughout the RP (green) and act as a chemical defense mechanism. Scale varies with perspective. Edges of the gray wood cube, 650 μm.

The living cells (parenchyma) in the secondary xylem of woody plants can provide a dynamic response to xylem infection and mechanical damage, and their role is critical to our understanding of tree defense systems. They make up a living tissue that forms two distinct types of cambial derivatives, ray parencyma (RP) and axial parenchyma (AP). RP cells are derived from the xylem ray initials and are laid down horizontally elongate (procumbent ray cells) in most cases; erect or upright ray cells are often present in species with multiseriate rays and are speculated to form an intermediary between procumbent ray cells and AP or vessels (Höll, 1975; Sauter and Kloth, 1986). AP cells are produced via the fusiform initials and are orientated longitudinally (Spicer and Groover, 2010). RAP (ray and axial parenchyma) cells are irregular in shape with gradations running between cubic and cuboid, depending on their function, and have, in most cases, thin walls. Although RP and AP run perpendicular to each other, they form a highly complex interconnected three-dimensional network providing a living continuum that is interspaced among the dead libriform fibers and water conducting cells of the secondary xylem, most notably, for the latter, the vessel elements and imperforated tracheary elements (including the fiber-tracheids) of angiosperms and the tracheids of gymnosperms (**Figure 1**). While RAP cells are the most abundant living cell types found in angiosperms, it is important to note that ‘living’ fibers are also present in many angiosperms (e.g., Araliaceae, Salicaceae, Sapindaceae; Esau, 1953; Fahn and Leshem, 1962; Wolkinger, 1970, 1971; Pratt, 1974). The presence of them tends to feature more when AP is absent or rare, while both cell types have been shown to function similarly (Wheeler et al., 2007; Yamada et al., 2011; Plavcová et al., 2016). However, living fibers will not be included under the umbrella of AP, as they are fibers and also function as them mechanically. Although living fibers are presumably influential in defense, little is known about their functions in relation to this, so they will not be addressed any further in the review.

The operation of RAP, both independently of each other and as a continuum, is largely not well understood. Similarly, the physiological and structural implications of the huge range of anatomical diversity between species represent a significant gap in our understanding of plant function. Where recent work has made some strides toward revealing the functional significance regarding the amount of AP in woody plants (Martínez-Cabrera et al., 2009; Zheng and Martínez-Cabrera, 2013; Ziemińska et al., 2013, 2015; Morris et al., 2016), very little is known about their spatial arrangements (Morris and Jansen, 2016). Conversely, much is understood about their role in storage and transport of assimilates (Evert and Eichhorn, 2006; Plavcová et al., 2016). However, their functional relationship with the dead conducting cells is less certain, as well as to what degree they contribute to long distance water transport in stems, or the role they play in defense against pathogens. It is important to tread carefully when partitioning the functions between parenchyma cells types, as many of the functions are performed by both RP and AP, while other more specialized functions are carried out separately by either of the two parenchyma types. Ultimately, they are all interlinked to some degree through a continuum via plasmodesmata connections between cells within the xylem matrix and their collective link to the phloem. For instance, the mechanical role of living cells is commonly accepted to be more closely related to the RP cells, where, owing to their orientation, they increase radial strength (Beery et al., 1983; Burgert et al., 1999; Burgert and Eckstein, 2001; Reiterer et al., 2002); however, AP cells also play a mechanical role through turgor pressure (Niklas, 1992; Chapotin et al., 2006). For most woody species the fibers or ‘living’ fibers assume the mechanical role. In some species (e.g., *Acer pseudoplatanus*) AP cells are even more strongly lignified than wood fibers (Schwarze et al., 2000b). Moreover, the roles of RAP as a whole in relation to tree hydraulics and its involvement in defense against decay spread by pathogens cannot easily be differentiated, as they are entwined. It is speculated that RAP indirectly prevents the spread of decay by keeping air-filled conduits isolated (Rayner and Boddy, 1988; Schenk et al., 2008), by refilling of conduits (Brodersen et al., 2010; Knipfer et al., 2016), or by keeping water-flow continuous through a buffering effect via capacitance (Meinzer et al., 2009; Jupa et al., 2016). Fungi flourish when oxygen is available, enabling them to use air-filled conduits as a kind of ‘highway’ for their growth and, inevitably, decay spread within the xylem (Baum and Schwarze, 2002; Yadeta and Thomma, 2013; Pouzoulet et al., 2014). Other roles of parenchyma that are directly or indirectly related to tree defense include: (1) storage of NSCs, while acting as a fuel for new growth along with cell metabolism and repair, NSCs also play a key role against drought stress (Rosas et al., 2013; O’Brien et al., 2014, 2015), and are pivotal against damage (including pathogens) as the carbon is used to build polyphenolic compounds and suberin, which are toxic to microbes (Hillis, 1977; Shain, 1979, 1995; Magel et al., 1994); (2) transition of sapwood into heartwood, where the living cells of RAP die and undergo chemical changes in the process that impregnate the heartwood with microbe resistant toxic extractives (Schwarze et al., 2000c; Spicer, 2005; Spicer and Holbrook, 2007), while the contact cells exude tyloses or gels (sometimes called plugs or gums) into the vessels, particularly by RP (Chattaway, 1949); and (3) the lignification of the secondary cell wall of RP, which, while providing biomechanical support, also imparts with a greater resistance to microbial penetration.

A clear understanding of the role of living cells in woody stems is still lacking, partly from a shortage of experimental studies of RAP in wood, which would give a stronger basis for functional hypotheses regarding RAP. While the bark is an integral part of tree defense (Franceschi et al., 2005; Paine et al., 2010; Ferrenberg and Mitton, 2014; Rosell, 2016), forming a continuum with the xylem (Spicer, 2014), our review paper will focus exclusively on the role of RAP of the secondary xylem in defense against fungal pathogens. The theme of the review principally focuses on decay-causing fungi (mostly Basidiomycota), while at times referencing Dutch elm disease, an important vascular wilt fungus that is examined in case studies using the CODIT model (Shigo, 1984). Although important, vascular wilt disease and the role of RAP is a subject by itself and should be treated in a separate paper. This review aims to outline what we currently know along with the directions of research we need to take to understand the role of RAP in defense in greater depth. Classical works will be examined, together with more current studies in order to thoroughly investigate wood structure-function in relation to tree pathology and thus provide a broader consensus of the role of RAP in tree defense. The sections will include: (1) a re-examination of the CODIT model with respect to the role of RAP, (2) RAP fractions and their spatial distributions in the sapwood in relation to defense, (3) fungal types and the interaction between decay fungi and RAP, (4) decay strategies used by fungi to overcome host strategies of both the sapwood and heartwood, and finally (5) a look at the relationship between the longevity of the plant and growth rate in relation to RAP and defense.

## RAP and the Codit Model – A Framework

Compartmentalization of Decay in Trees is a concept to explain how decay is sectioned off or compartmentalized resulting in the ability to stop or restrict its spread (Shigo, 1970, 1976, 1979, 1984; Shigo and Hillis, 1973; Shigo and Marx, 1977; Merrill and Shigo, 1979). The model derived from previous studies into decay and boundary setting in trees (Hartig, 1878; Frank, 1895; Küster, 1913; Hepting, 1935; Hepting and Blaisdell, 1936). Although met with criticism (Boddy and Rayner, 1983), the conceptual framework of CODIT is most elegant in its simplicity, providing a strong basis by which to understand how large and highly compartmented organisms defend themselves. Related models that look at tree compartmentation, but are focused on water transport in vessel-bearing angiosperms, include: hydraulic segmentation (Tyree and Zimmermann, 2002), hydraulic sectoriality (Orians et al., 2002, 2004; Zanne et al., 2006), hydraulic redundancy (Ewers et al., 2007; Schenk et al., 2008), and spatial arrangement of conduits (Loepfe et al., 2007; Martínez-Vitalta et al., 2012). These concepts are all similar, but functional at different macroscopic and microscopic levels, from whole plant down to organ and cell level.

**FIGURE 2:**
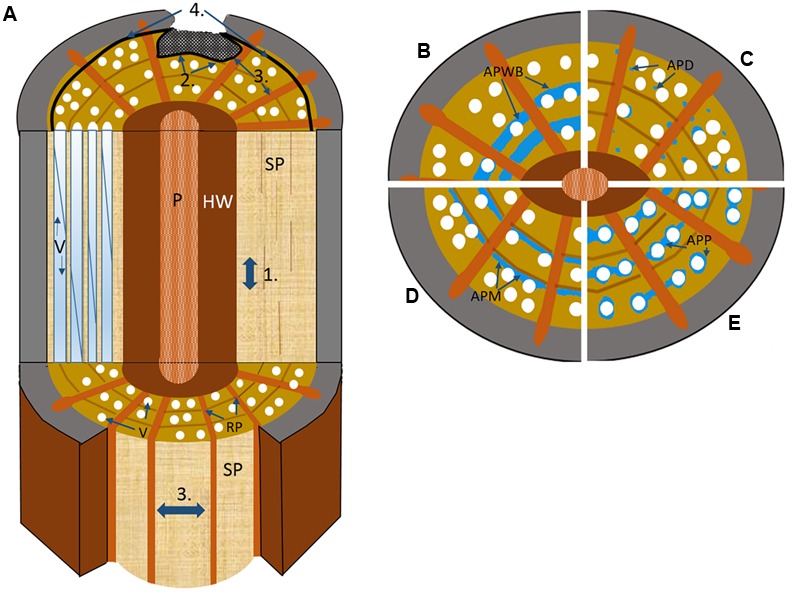
**Schematic drawing illustrating the four walls of the CODIT model. (A)** Wall 1: limits the up or downward movement of decay through the plugging of the vascular elements by RAP via tyloses or gels; Wall 2: limits the radial movement of decay (both inward and outward) at the growth ring boundary, where in addition to strongly lignified fibers, apotracheal marginal AP contributes, and possibly the wide bands of AP in many tropical species in the absence of growth rings; Wall 3: the strongest wall at the time of wounding, which limits the lateral movement of decay spread through chemical alteration of ray parenchyma (RP) along with the plugging of vessels via the contact RP cells; Wall 4: the strongest wall and the only wall formed after wounding, forms a chemically distinct and impervious barrier that separates new wood from the old infected wood to its interior. P, pith; SP, sapwood; RP, ray parenchyma; V, vessel. **(B–E)** the possible contributions different AP patterns make to defense based on cross-sectional wood representations divided into quarters, with **(B)** axial parenchyma wide banded (APWB) often present in the absence of distinct growth rings in tropical species, where it likely functions as part of wall 2, **(C)** axial parenchyma diffuse (APD) found predominately in temperate species where AP is often seen to be scattered more randomly and in low amounts, which could indicate that these species are poor at forming reaction zones (wall 1–2) with RP (wall 3) possibly taking on a greater defensive role, **(D)** axial parenchyma marginal (APM) forms an important defensive component alongside the highly lignified fibers at the growth ring boundary, and **(E)** axial parenchyma paratracheal (APP), more often present in large-vesselled species of tropical origin.

The CODIT model is made up of four ‘so-called’ walls in two-parts (**Figure 2**), which are conceptual and explain the anatomical divisions or compartments that exist in woody plants at the time of wounding (part I), and divisions built after wounding (part II) by the plant to limit the spread of air or decay progressing into new wood. Part I, the reaction zones, includes walls 1–3, while wall 4 (the barrier zone), makes up part II of the process. The walls move chronologically in order of effectiveness, with wall 1 being the weakest wall and wall 4 the strongest. RAP plays a fundamental role in all four walls of CODIT by forming reaction zones (wall 1–3) at the margin of infection through: (1) the production and release of microbe resistant polyphenolic compounds, from where they have been found to infiltrate the intercellular spaces between RAP and the cell lumina of fibers (Woodward, 1992; Pearce, 1996; Schwarze and Fink, 1998); (2) through tyloses – balloon-like sacks that create a physical barrier that render the vessels non-functional (Zimmermann, 1979; Bonsen and Kucera, 1990; Schmitt and Liese, 1990, 1994; Bonsen, 1991); and (3) through suberization – the deposition of a water-repellent fatty layer that is also toxic to microbes owing to its phenolic constituent (Biggs, 1987; Schmitt and Liese, 1993). RAP forms new callus in the immediate vicinity of the injured area (Wargo, 1977; Shigo, 1984), or wound-induced traumatic resin ducts in the conifer families Pinaceae and Cupressaceae (Phillips, 1948; Hudgins and Franceschi, 2004). In addition, beyond the barrier zone (of wall 4), a new chemically altered living tissue, known as wound wood (callus tissue and altered wood), is produced by the cambium prior to the resumption of “normal wood” (Eyles et al., 2003). An important amendment to the definition of CODIT was proposed by Boddy and Rayner (1983), and was then further developed by Liese and Dujesiefken (1989, 1996), where the latter authors rightly stated that the ‘D’ should preferably stand for ‘Damage’ or ‘Dysfunction’ or at least be an alternative substitute for ‘Decay’. This amendment to the terminology lies in the fact that the walls do not only and specifically react against fungi, but do so against any desiccation-inducing threat, including biotic factors such as herbivores and insects, and abiotic factors, for example general breakage caused via wind and snow.

The weakest construct of CODIT is wall 1 (**Figure 2A**), which is both static and anatomical (i.e., the branch protection zone) in its nature as well as dynamic (i.e., reaction from the living cells), and limits upward and downward movement of decay in the sapwood. It is the least effective of the four walls and does not compromise the integrity of the vertical system, with particular reference to the hydraulic system. There is a trade-off here, where to limit decay spread up or down the stem, or the vertical movement of mycotoxins via the transpiration stream, the conduits must be sealed off with tyloses or gels, originating from contact parenchyma (RP or AP in direct association with a conduit), at the expense of continued water and solute movement (Schmitt and Liese, 1990; Clérivet et al., 2000; Sun et al., 2007). In other words, to be more effective than it needs to be would be ‘surplus to requirement’ and could indirectly be the cause of tree death through hydraulic failure. The integrity of the hydraulic conduit system must be kept intact, as this is the most vulnerable component of the xylem. When fully functional, high moisture levels are arguably the tree’s greatest defense (Boddy and Rayner, 1983; Rayner and Boddy, 1988; Baum and Schwarze, 2002). In elm (*Ulmus* spp.) infected with Dutch elm disease (*Ophiostoma novo-ulmi*), localized blockage of vessels is so efficient, owing to production of toxins by the fungus, that vertical transport to the canopy is often blocked, contributing significantly to tree death (Newbanks et al., 1983; Ouellette et al., 2004; Solla et al., 2005). Vascular blockage by gels containing pectins along with suberin arising from parenchyma was found to occur quite rapidly in the secondary xylem of *U. americana* (American elm) after inoculation of *Ophiostoma novo-ulmi* (Rioux et al., 1995). The hydraulic redundancy model (Ewers et al., 2007; Schenk et al., 2008) explains how these trade-offs can be affected by the degree of “inter-connectivity” between vessels in plants, where species with an integrated redundancy (high degree of inter-connectedness) create alternate pathways around blockages arising from tyloses or disease. However, increased xylem connectivity and hydraulic redundancy comes with a significant risk: both pathogens and emboli can follow the same network of linked conduits, thereby increasing the likelihood of systemic pathogen spread or catastrophic hydraulic failure. How plants have evolved to balance the costs and benefits of xylem interconnectivity is still largely unexplored, but this balance clearly plays an important role in plant defense within the CODIT framework. Dutch elm disease is an exception in that it is defined as a vascular wilt and does not cause decay, a pertinent point when the original interpretation of CODIT (compartmentalisation of ‘decay’ in trees) does not typically include vascular wilt diseases. However, Shigo et al. (1980) along with Shigo and Tippett (1981) have used CODIT to describe the nature of tree responses to Dutch elm disease, and the concept has supported various wilt diseases more widely since (Pouzoulet et al., 2014).

**FIGURE 3:**
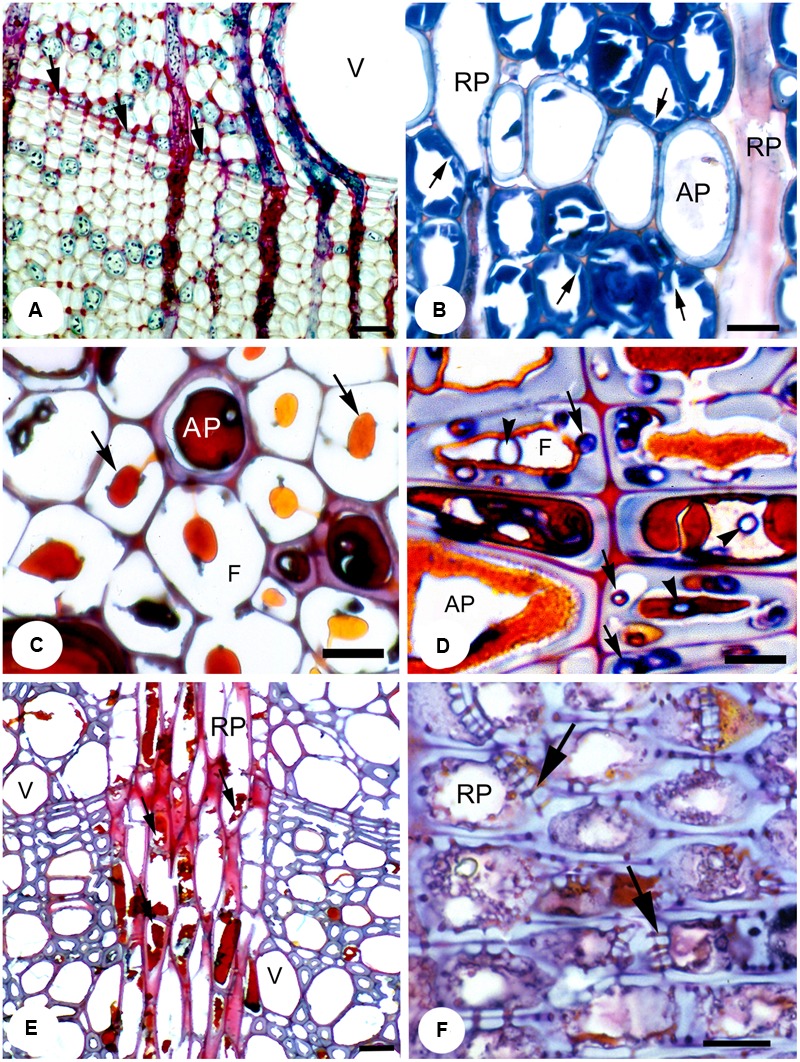
**A range of temperate species showing the effects of fungal pathogens on RAP. (A)** Transverse section (T.S.) showing apotracheal marginal parenchyma (arrows) in pedunculate oak (*Quercus robur*), which forms part of wall 2 of CODIT, together with the strongly lignified fibers. Bar, 50 μm. **(B)** pedunculate oak incubated with *Laetiporus sulphureus*. The secondary walls of the libriform wood fibers show numerous clefts resulting from fungal enzymatic activity (arrows). Bar, 20 μm. **(C)** T.S. of a reaction zone in common beech wood (*Fagus sylvatica*) artificially incubated with the soft rot *Kretzschmaria deusta* showing the persistence of brownish polyphenolic deposits (arrows) and the compound middle lamellae in otherwise strongly degraded wood; polyphenols derived from an AP cell. Bar, 10 μm. **(D)** T.S. of London plane (*Platanus × hispanica*) wood naturally infected with *Inonotus hispidus*. Abundant polyphenolic deposits are apparent within the lumen of AP and adjacent fiber tracheids. Degradation of cell walls occurs only where the hyphae have escaped adverse conditions by growing in the cell wall (arrows). In contrast, hyphae within the lumina (arrowheads) are arrested and have no visible effect on the cell wall structure. Bar, 10 μm. **(E)** T.S. of a reaction zone (wall 3 of CODIT) in London plane wood naturally infected with *Ganoderma adspersum*. Most polyphenols within RP cells have been degraded (arrows) by the fungus. Bar, 50 μm. **(F)** Radial longitudinal section of RP cells within a reaction zone of large-leaved lime (*Tilia platyphyllos*) wood showing the presence of polyphenolic deposits and occluded simple pits (arrows) in RP. Bar, 10 μm. Reproduced from Schwarze (2008).

Wall 2 (**Figure 2A**), a medium strength response, acts by halting the spread of decay inward toward the pith. It usually comes into effect after wounding of the cambium, and is part of the existing xylem structure in tree species with distinct growth rings. The growth-ring boundary in trees that undergo seasonality makes up a static or physical wall (unable to chemically respond to pathogenesis), where the highly lignified cell walls of the fibers act to inhibit radial movement, as lignin is a highly durable polymer (Shigo and Marx, 1977). In fact, fiber wall thickness is one of the best predictors for hydraulic compartmentation (independent hydraulic redundancy) in shrubs (Schenk et al., 2008). This physical barrier is continuous except when interrupted by the ray system at more or less regular intervals. Aside from RP providing a living and dynamic defensive component to an otherwise static barrier, AP also plays a large role in the defensive properties of wall 2 (Biggs, 1986b; Schwarze and Fink, 1997; Schwarze et al., 2000c, 2003; Schwarze, 2007). Arranged in bands stretching between rays and formed at the end of a growth increment (terminal parenchyma) or at its beginning (initial parenchyma) are the apotracheal AP cells that form more or less continuous lines of varying widths, collectively called marginal parenchyma (Jane, 1934; Hess, 1950; IAWA Committee, 1989; Wilczek et al., 2014). They act to support the thick walled fibers formed at the end of the growth season (**Figures 3A,D**). However, these studies are biased toward tree species of the temperate regions, where tree rings form as a natural part of a seasonal climate. In the lowland tropics, where trees rings in species are not clear or, in many cases, are completely absent, it could be speculated that the high occurrence of wide-banded AP (parenchyma running between rays ≥three cells wide) are taking on the functional role of defense in the absence of highly lignified fibers (**Figure 2B**). An analysis of 834 species with wide AP bands within the InsideWood database (Wheeler et al., 2007), showed a remarkably strong correlation between wide-banded AP and absent or indistinct growth increments (i.e., in 89% of the species; Morris and Jansen, 2016). The advantages or disadvantages of having either static or physical partitioning between growth increments or a dynamic barrier composed of living cells, or a combination of both, remain unclear. An interesting result from a large meta-analysis looking into plant defense theory, was the trade-off between the deployment of secondary metabolites in dynamic plant responses and physical or structural barriers already in place as part of an anatomical suite, with the latter found to be far more effective (Carmona et al., 2011). Although this study concentrated wholly on insects and herbivores and not pathogens, it may explain why such wide bands, by way of compensation, are replacing growth rings in many lowland tropical trees compared to the trees rings in temperate species, which often have only narrow marginal bands.

The last wall of part I of the CODIT model, or wall 3 (**Figure 2A**), is the strongest wall in place at the time of wounding, which restricts pathogenesis spreading in a lateral direction (Shigo, 1984). It is composed completely of RP, although conifers often have ray tracheids at their tips, which are dead at maturity (Singh et al., 2006). These walls extend outward from the heartwood-sapwood boundary, forming a physical and dynamic barrier and, thereby compartmentalizing much of the stem into discreet units in species where the walls also extend for significant axial distances (**Figures 1–3**). Wall 3 can have widths ranging from uniseriate (one-cell wide) to multiseriate (≥3 cells wide; IAWA Committee, 1989; Carlquist, 2001), with conifers having, in most cases, the former, where every RP cell has some contact with a tracheid. RP typically has lignified secondary cell walls (Evert and Eichhorn, 2006), although the amount of lignin varies between species and plant growth forms. For instance, lianas have RP cells that are thin-walled, which is speculated to allow for increased twisting without rupture (Schenck, 1893; Haberlandt, 1914; Sieber and Kučera, 1980; Gartner, 1991; Putz and Holbrook, 1991). In *Liquidambar*, however, the rays have an even higher specific gravity (relative density) than the surrounding fibers (Taylor, 1969), which may explain why their cell walls are resistant against a number of important fungi to a high degree, while the fibers are easily digested (Yilgor et al., 2013). The high anatomical variation in the structure of rays (Chattaway, 1951), from their height through to their width and connectivity with AP, could also help evaluate the effectiveness of wall 3 between species. For example, invasiveness of the basidiocycete *Inonotus hispidus* on London plane (*Platanus × hispanica*) and common ash (*Fraxinus excelsior*) varied a great deal owing to the differing anatomical makeup of these two tree species, where the rays of the latter were easily breached by the fungus (Schwarze et al., 1995a). The RP cells of *Ailanthus altissima* showed a similar resilience to those of the London plane, with only minor degradation of the cell walls by *I. hispidus* observed (Koyani et al., 2010, 2015). Along with polyphenolic compound activity in RP during defense, suberin, formed as a reaction to cell death, is typically found in the bark of trees, was observed in the reaction zone of wounded RP in a range of conifers and deciduous broadleaf temperate species (Pearce and Rutherford, 1981; Biggs, 1987), demonstrating how suberin, a fatty sealant, can be so effective in delimiting the expanse of necrotic tissue, although it is also highly energy demanding owing to its molecular complexity. Of importance is that once ray cells form reaction zones against the movement of fungal hyphae, they die, and are similar to heartwood in coloration and function. Dead parenchyma plays a critical role in defense against decay-causing fungi.

The final wall, referred to as wall 4 (part II), often called just as the ‘barrier zone’ (**Figure 2A**), was first coined by Shigo and Tippett (1981) and is defined as a zone of distinctive rings of parenchyma cells produced by fusiform initials in response to injury, which serves to separate necrotic tissue from new wood formed after the barrier zone is in place (Tippett and Shigo, 1981). However, distinctive formations of parenchyma in response to vascular pathogens had been noted before the invention of the term (Buisman, 1935; Schoeneweiss, 1959; Banfield, 1968). The parenchyma cells of the barrier zone go through a series of changes before ‘normal wood’ can resume development upon successful compartmentalization of the wound. Likened to terminal parenchyma laid down at the beginning of a new growth increment, once differentiated, the new AP accumulate starch, which is replaced by polyphenols and, in many cases, a suberin rich cell wall, giving the barrier zone its unique rich brown color comparable to that of the reaction zones formed early during the CODIT process (Tippett and Shigo, 1981). In *U. americana* infected with *Ophiostoma novo-ulmi* annual shoots and small branches were found to have suberized parenchyma cells in their barrier zones, which formed air tight layers protecting the newly developed cells beyond wall 4 of CODIT (Rioux and Ouellette, 1991a,b). Although the wall is chemically strong (protective), it is structurally weak owing to the reduced numbers of vessels together with a decrease in lignin content of the cell walls, which was found to be the case in a number of temperate species including *Acer saccharum, Betula alleganiensis* and *Fagus grandifolia* (Rademacher et al., 1984). The same authors found that in *Acer saccharum* parenchyma made up a remarkable 95% of the barrier zone, with only 5% vessels and no fibers.

A well-developed (rarely enclosing the entire stem) barrier zone is only built as a last resort, usually when the other walls have failed to contain the decay spread. This makes sense due to the high energy cost of building a barrier. Once wall 4 completely envelops the circumference of the stem or root, untapped storage sinks of NSCs are no longer accessible for tree growth and function, as the symplastic pathway for metabolite movement has been disconnected (Moore, 1978; Mulhern et al., 1979; Pearce and Rutherford, 1981; Shigo, 1982; Pearce and Holloway, 1984; Bonsen et al., 1985). In order to continue active growth the new storage RAP cells laid down to the exterior of the barrier zone must compensate for this loss, initially at the expense of fibers and vessels. Older NSC reserves stored in RAP of previous years (up to decades in some cases) can be plentiful after successful growth periods where supply exceeds demand, thus storing reserves for future uncertainties (Kozlowski, 1992; Gleason and Ares, 2004; Richardson et al., 2012; Hartmann and Trumbore, 2016). Indeed, studies have shown that new growth is in part fuelled by NSCs derived from more distal parts of the stem and roots, where it is pooled with more recent NSCs (Keel et al., 2007; Carbone et al., 2013; Mildner et al., 2014). Of note is where successive barrier zones are formed after failure to supress the decay, thus placing overburdening demands on the current growth years NSC supplies (Tippett and Shigo, 1981). Interestingly, regarding the effectiveness of the barrier zone, there appears to be a trade-off between it and the reaction zones (walls 1–3), where trees with a strong barrier zone tend to have weak reaction zones, with most reaction zones eventually being breached by even moderately invasive fungi, but successfully compartmentalized by the barrier zone [e.g., in *Tilia platyphyllos* (Baum and Schwarze, 2002)]. Support for the latter was found in a study of tropical species by Romero and Bolker (2008), which is discussed later in the review; however, research on a range of species would be required to establish that a pattern does indeed exist.

Included under the umbrella of wall 4 (any new tissue formed after injury), is the generation of new parenchyma cells after a wound has been created, forming a callus tissue (not wound wood in this case) that seals the damaged region and restricts an ingress of both air and pathogens. Often, depending on the depth of the wound, tissue repair occurs through generation of immature parenchyma (totipotency), irrespective of a ‘typical’ barrier zone, with the latter being formed via the cambium only (Shigo, 1984; Eyles et al., 2003; Frankenstein et al., 2005; Schmitt et al., 2006). An unusual response was found in *Adansonia digitata* (baobab tree) during the growing season, where the exposed wound, after branch removal, was sealed exclusively through RAP proliferation from just inside the damaged outer layer, providing evidence that for this species all parenchyma from pith through to the secondary xylem can dedifferentiate, while the cambium mostly remains defensively inactive (Fisher, 1981). The depth of the wound, whether it is confined to the bark, or extends into the xylem, plays a large part in deciding what kind of cellular response is involved. For instance, if the wound enters no deeper than the inner bark or just beyond the cambium, the wound can be sealed via mostly undifferentiated immature parenchyma cells that have yet to develop secondary walls, with the new tissue being referred to as ‘surface callus’ (McDougall and Blanchette, 1996; Dujesiefken et al., 2001; Stobbe et al., 2002). The isodiametric parenchyma cells of the callus tissue were characterized as having large vacuoles and thin primary cell walls, along with many intercellular spaces in a range of species (e.g., *Hibiscus, Hevea brasiliensis, Populus* spp.; Sharples and Gunnery, 1933; Brown and Sax, 1962). In these species, an influx of phenolic substances followed, along with the formation of suberin rich walls, prior to the development of wound periderm (outer callus) and wound cambium. As well as through healing a wound through immature parenchymatous inner bark tissue, shallow wound repair can also occur through dedifferentiation of phloem parenchyma.

Normally if the wound extends beyond the bark and into the secondary xylem, the RAP farther inward cannot dedifferentiate, as they already have well-developed secondary cell walls. Instead, a reaction zone (walls 1–3) is formed just in the vicinity of the wound. Evidence of the latter was found in *Betula* spp. and *Tilia* spp. where reaction zones (referred to as a boundary zone by the authors) formed. The reaction zones prevented air and microorganisms from entering through the occlusion of water conducting cells by tylosis, and the deposition of wound-associated polyphenols via modified pits of the contact RAP into the fibers and vessels (all cells fully differentiated at time of wounding; Schmitt and Liese, 1992; Schmitt et al., 2007; Schmitt and Koch, 2009). The barrier zone initiates laterally and vertically from the peripheries of the wound via the ‘intact’ cambium. Working together, both reaction zones and the barrier zone can completely compartmentalize the wounded area from the new tissue formed later. The important point to make here is that wall 4 does not comprise exclusively of the barrier zone formed via the cambium, but another type of barrier zone may be designated to callus produced by immature parenchyma at the surface of a shallow wound in the inner bark or just inside the sapwood (Shigo, 1984).

## RAP Fractions, Spatial Distribution and Function

Although attention has been given to the living cell’s involvement in detection and response to decay or injury, none of the major studies carried out in the latter part of the 20th century addressed the anatomy of RAP, including their abundance across species, or their arrangement, in any great detail. Some reports incorrectly suggested that species had on average less than 10% living cells (Hillis, 1977; Boddy and Rayner, 1983). As a result, the contribution of living cells as a defense component was considered to be relatively small when compared to more indirect mechanisms (Boddy, 1992). This estimate should not be applied across a spectrum of trees owing to the huge variation between species, and could therefore not be used as part of a general tool on which to base a defense model. It is also biased toward temperate species, as comparatively few studies on tree defense have been conducted in the tropics. Along with defense, when considering all the other functions that RAP are hypothesized to be involved in, the percentage fraction of the sapwood they take up together with the spatial distribution (of AP in particular) should make a considerable difference to a tree’s overall potency as a defense system. However, this theory may well depend on climate and phenology, factors which are likely to influence the behavior of RAP and the roles they fulfill.

In general, while RP cells, with the exception of rayless species (Carlquist, 2001), are more uniform and consistent in their amount (between 4 and 20%) and spatial arrangement between taxa (Fahn et al., 1986; Morris et al., 2016), AP cells, on the other hand, vary greatly in both the proportion (between ≤1 and ≥30%) and arrangement (Wheeler and Baas, 1991; Alves and Angyalossy-Alfonso, 2002), a condition that is taxon specific making it very useful for the identification of hardwoods (IAWA Committee, 1989). Morris et al. (2016) showed that the percentage of RAP within the xylem is associated with increasing mean annual temperature, with RAP percentage rising toward the tropics; especially AP, which showed a sharp rise in areas with a mean annual temperature above 16°C. Overall, RAP proportions can be as high as 88% in *Adansonia* spp. (Chapotin et al., 2006), and as low as 3.4% in *Thuja occidentalis* (Wagenführ, 2007). When considering the huge variation in RAP amounts, trying to pin-point to what extent selective pressure is defining CODIT and defense when we acknowledge other important hypothesized functions of RAP such as their role in capacitance, embolism repair, NSC storage (Plavcová et al., 2016), a biomechanical role, and possibly a role in controlling hydraulic conductivity of the xylem (Nardini et al., 2011) is a real challenge for plant scientists, where many trade-offs are likely at play. In shrubs, the degree of contact between AP and vessels decreases with rainfall, while contact of RP with vessels shows the opposite relationship with rainfall (Martínez-Cabrera et al., 2009). The spatial arrangements of AP are also influenced by climate. Apotracheal arrangements, such as diffuse or diffuse-in-aggregates (little or no contact with vessels) are more common in temperate regions (**Figure 2C**), while paratracheal arrangements (groups of AP surrounding vessels and often linking vessels and rays) such as aliform and confluent AP, together with banded AP, are more frequent in the tropics (Wheeler and Baas, 1991).

An interesting study of seven Bolivian tree species by Romero and Bolker (2008) showed that species which demonstrated poor compartmentalization (walls 1–3), compensated for this by rapid wound closure (living cells formed after injury; wall 4 of CODIT) due to thicker bark and wide dilating rays present in the phloem. For instance, *Ceiba speciosa* showed rapid and complete bark wound closure while having traits that favored xylem decay spread, whereas the opposite trend was found in *Pseudolmedia laevis*, with the latter species having more effective walls 1–3 of CODIT. A trade-off between the phloem and xylem is apparent, where both together are unlikely to be equally effective in combating injury (Biggs, 1986a; Romero and Bolker, 2008). The fast wound closure in the bark of these species could seal off air channels that pave the way for decay propagation, lowering the requirement for a strong defensive strategy in the sapwood. However, the study in Bolivia was carried out on a small sample size, so precautions must be taken when drawing conclusions here. The dedifferentiation of RAP shown after wounding (large branch removal) in the highly parenchymatous *Adansonia* (baobab) also provides evidence supporting a trade-off between wall 4 and the reaction zones (walls 1–3), where the capability of RAP cells to divide and rapidly close wounds overcomes a necessity for RAP to produce reaction zones and die in the process (Fisher, 1981), or an adaptation (pachycauls and lianas) to having mechanically weak stem structures that are easily damaged (Dobbins and Fisher, 1986; Fisher and Ewers, 1989, 1991). This adaptive approach could be regarded as an offensive rather than a defensive strategy.

In species of cooler temperate climates, where overall RAP abundance among species is lower than in the tropics, the role of parenchyma as a defense tissue may take on higher priority compared to other functions. RAP was shown to be effective against brown rot fungi (Schwarze et al., 2003) where species with higher RAP fractions in temperate regions might be more effective in forming reaction zones (walls 1–3 of CODIT). In agreement with this, numerous studies have shown that the amount of RAP is higher in trees of temperate origin that have recovered from pathogenesis (Tippett and Shigo, 1981; Schmitt and Liese, 1990; Arbellay et al., 2010, 2012). It has been noted that for conifers, where the proportion of RAP is generally low (AP in particular), suberization responses were poorly developed (Pearce, 1990).

Species with fewer AP cells might benefit from having smaller conduit sizes, which is the case in trees of temperate origin (Zanne et al., 2014; Morris et al., 2016; Hacke et al., 2016). Smaller conduits can maintain hydraulic integrity with greater ease, as there is less opportunity for emboli formation due to having fewer and smaller pits (Sperry and Saliendra, 1994; Wheeler et al., 2005), and therefore, a reduced chance for fungal development (Martín et al., 2009; Pouzoulet et al., 2014). Also, it has been shown in *Ulmus* spp. infected with *Ophiostoma novo-ulmi*, that small vessels (in length and width) are more quickly clogged by tyloses owing to their smaller lumen areas (wall 1 of CODIT) arising from the contact parenchyma (McNabb et al., 1970).

## RAP and Fungal Types

There are three main fungal decay groups recognized, each with a different mode of action: (1) brown rot, (2) white rot, and (3) soft rot. An overview of the role of RAP in defense against decay fungi in various temperate tree species is provided in Supplementary Table S1.

Approximately 80% of brown rot is associated with conifers (division Pinophyta) where it causes the breakdown of the S_2_ layer of cell walls through the release of hydrogen peroxide, giving the decayed wood a brown appearance (Lodge, 1993). Moreover, oxalic acid and iron ions are released, leaving the S_3_ layer and the lignin-rich middle lamella relatively unscathed, demonstrating a preferential decomposition of hemi-cellulose and cellulose (Koenigs, 1972, 1974; Rayner and Boddy, 1988; Green and Highley, 1997; Schwarze, 2008). Because of this pattern, latewood tracheids (in conifers) and fibers (in angiosperms) are mostly left intact until an advanced stage of decay. Interestingly, brown rot fungi do not degrade RAP, at least not until an advanced stage, as shown in a study by Schwarze et al. (2003), which found that the cell walls of RAP remained intact. This finding is perhaps due to the absence of radial fibril orientations in the secondary walls, where such a structure could allow digestive enzymes from the fungus to diffuse into the S_2_ layer more rapidly (**Figure 3B**). Schwarze et al. (2003) also found a correlation between RAP amount and weight loss in trees, due to brown rot. The highest weight losses were recorded in species with the lowest RAP fractions, such as in Norway spruce (*Picea abies*) and birch (*Betula* spp.), where RAP accounted for between 4–10% (Wagenführ, 2007), while the lowest weight losses were found in species with much higher RAP proportions, such as in oak (*Quercus* spp.) and black locust (*Robinia pseudoacacia*) with RAP levels of between 35–40% (Wagenführ, 2007). The latter might suggest why so few angiosperms are infected by brown rot fungi, as conifers have lower RAP fractions, in particular AP. Also, brown rot fungi are rare in most parts of the southern hemisphere, where conifers are less prevalent and where angiosperms have, on average, much higher RAP fractions than temperate tree species, with the exception of temperate southern hemisphere regions, i.e., Patagonia (Morris et al., 2016). Regarding the CODIT model, brown rot fungi would be less effective in lateral (wall 3) directions of species with multiseriate rays, and in longitudinal and radial directions of species with high AP fractions, notably marginal AP, which run in bands between rays (**Figure 3D**). Brown rot fungi that occur both in conifers and angiosperms include *Laetiporus sulphureus*, which is found on *Taxus baccata* (common yew), *Quercus* spp, *Castanea sativa* (sweet chestnut), and *Robinia pseudoacacia* (Burdekin, 1979).

White rot is caused by species of fungi from both the Basidiomycota and the Ascomycota, and is most predominant within the angiosperms. The high anatomical variation and more abundant and diverse RAP patterns of angiosperm wood could indicate why white rot is so prevalent globally. White rot is further divided into two broad divisions: selective delignification (**Figure 3E**) and simultaneous rot. The former degrades lignin preferentially prior to hemicellulose and cellulose, using a range of oxidative enzymes, including laccase, tyrosinase and peroxidase (Schwarze et al., 2000c; Schwarze, 2008), while the latter degrades both lignin and cellulose together at approximately equal rates, where all substances of the cell wall are digested (Liese, 1970). In contrast to the brown rot, white rot fungi of the selective delignification type are able to destroy the cell walls of RAP (and possibly ‘living’ fibers) with ease during the early stages of delignification, whereas the fibers (libriform) are left intact. This strategy was observed in *R. pseudoacacia* artificially incubated with the white rot, *Perenniporia fraxinea* (Schwarze, 2008).

Regarding simultaneous white rot, there is no marked preferential treatment for any cell type, although the guaiacyl lignin of vessels and RAP appears to be more resistant compared to the guaiacyl-syringyl phenylpropanoid combination in fiber cell walls (Wu et al., 1992; Schwarze et al., 2000b,c). An example of this was shown in the sycamore maple (*Acer pseudoplatanus*) by *Armillaria mellea*, where the cell walls of AP remained mostly intact, even where decay was advanced in other regions (Schwarze et al., 2000b). However, Bari et al. (2015) showed how RAP cells were completely destroyed by simultaneous white rot patterns of *Pleurotus ostreatus* and *Trametes versicolor* on Oriental beech (*Fagus orientalis*). This affirms that regardless of the type of rot the fungus is classified as, the pathogenic capability of the fungal species together with resistance of the host species (genetic capacity and its vitality at time on infection) plays a more central role in deciding how effective RAP are at either an early or advanced stage of decay.

In angiosperms, the interconnectivity between fibers, vessels, and RAP makes up a network that facilitates hyphal spread, especially hyphal growth through the rays, which exposes new areas of sapwood to pathogenesis (wall 3 of CODIT). The spread pattern of white rot fungi occurs via enzymes and direct hyphal penetration either through intercellular pits or through boreholes. The boreholes (also known to occur in brown rots) are small cavities formed in the cell walls and are caused by specialized cell-wall degrading hyphae (Bayliss, 1908; Nutman, 1929; Liese and Schmid, 1966; Liese, 1970; Schwarze et al., 2000a). Certain species of fungi, such as *I. hispidus* and *Meripilus giganteus* use RP cells effectively to breach the reaction zones of wall 3 (**Figure 3D**). Although the latter is a strong wall, once breached, the defense system must rely on the barrier zone of wall 4 to contain pathogenesis, thus bringing the plant’s defense system to the next level.

The final decay pattern is the soft rot, which occurs most extensively in the Phylum, Ascomycota, including the more unusual case of the facultative parasite, *Kretzschmaria deusta*, being classified as a white rot (**Figure 3C**). However, a facultative soft rot pattern has also been observed in the Basidiomycota, where a switch mechanism from the normal decay pattern to a soft rot is deployed; these include white rots (e.g., *Armillaria mellea, I. hispidus, I. dryadeus, M. giganteus*; Hartig, 1878; Schwarze et al., 1995b; Schwarze and Engels, 1998; Schwarze and Fink, 1998) and brown rots (e.g., *Fistulina hepatica*; *Rigidoporus crocatus*; Duncan, 1960; Schwarze et al., 2000a). With soft rot, the hyphae develop within the cell wall and follow the direction of the microfibrils, resulting in cavity formation in the form of oval or circular holes in the secondary cells (Savory, 1954; Courtois, 1963; Liese, 1964). The cavity formation of soft rot is analogous to the white rot fungi in that it results from the close proximity of the hyphae to the cell wall, which is not the case with brown rot decay. However, the soft rot pattern bears a strong similarity to brown rot decay in that it preferentially breaks down cellulose, leaving lignin mostly intact until a later stage of the decay process. Moreover, soft rot fungi, like brown rots, have difficulty breaking down RAP cell walls, possibly due to the absence of radial structures in their secondary walls (S_2_ layer), which are found in fibers and tracheids. Instead, RAP cells consist of mostly concentric lamellae (Zimmermann and Sell, 1997; Schwarze et al., 2000a), an arrangement that is mechanically more vulnerable, but less penetrable for the hyphae of soft rots, unlike the radial orientation of microfibrils in the secondary walls of fibers and tracheids. Also, the thin cell walls of parenchyma may deter soft rot fungi, as to develop suitably wide cavities requires more space (Courtois, 1963; Schwarze and Engels, 1998).

## RAP and Fungal Strategies

The defensive strategy undertaken by the host against a pathogen and the eventual outcome of that interaction is dependent on a range of factors, including (1) the genetic makeup of the tree species (Blanchette, 1992; Rayner, 1993), (2) the plant’s vitality at the time of pathogen entry, which is influenced by the age of the plant within the confines of its life history, the pH and nutrient status of the soil, and climatic conditions, (3) the status of beneficial symbionts, such mycorrhizal fungal networks, especially regarding soil borne pathogens (Azcón-Aguilar and Barea, 1997; Sikes, 2010), (4) phenology, especially in temperate tree species, and (5) the potency or virulence of the pathogen. For hardwoods in particular, the defense reactions at the time of wounding depend on the physiological activity and chemical constitution of RAP (Schmitt and Liese, 1993; Schmidt, 2006). For instance, high concentrations of polyphenolic compounds were found in a more resistant cultivar of grapevine to the fungus *Eutypa lata* (Rolshausen et al., 2010).

The behavior of RAP in defense is strongly influenced by the pathogen type. A vascular wilt fungus, which is largely vessel-confined, can be blocked and killed by chemical-coated tyloses originating from the contact cells of both RP and AP via half-bordered pits, with the mechanism being activated extracellularly from within the conduit (Yadeta and Thomma, 2013). In a revealing study by Rioux et al. (1998) it was found that secretion of pectin was associated with tylosis and gel formation in trees, which, prior to secretion, accumulated in the amorphous layer of contact cells (AP-conduit contact) along with the pit-membrane. The amorphous layer is located between the plasma membrane and the cell wall of all RAP cells that are in contact with vessels. Tylosis and gel formation clog the vessel completely through water-induced expansion presumably caused by the pectin (Rioux et al., 1998). Although the presence of pectic polysaccharides (based on immunolabelling techniques) in the amorphous layer is supported in a range of species (Fuji et al., 1981; Mueller and Beckman, 1984; Wisniewski and Davis, 1995), other more recent works found no evidence for pectins there (Plavcová and Hacke, 2011; Herbette et al., 2014; Klepsch et al., 2016). It could be that some species do not have pectin in the amorphous layer while others do, or pectins may only be present in the amorphous layer at certain times of the year. Also, it could be speculated that the presence or absence of pectin in the amorphous layer is strongly related to the ability of a plant to develop tyloses or gels. However, more research is required in this area, as we still know very little about the roles of the amorphous layer (Spicer, 2014).

Unlike the vascular wilts, wood decay fungi are not confined to the embolized vessels, where a number of strategies are used to breakdown wood, including: (1) heart rot, (2) active pathogenesis, (3) specialized opportunism, (4) desiccation tolerance, and (5) unspecialized opportunism (Rayner and Boddy, 1988). The heart rot fungi are specialized to cope with the low oxygen environment of the heartwood where due to the absence of living cells, there is no active defense response from the plant. However, the chemical compounds released from the parenchyma during their transition to heartwood serve as protection to varying degrees, depending on the plant species and their investment in heartwood protection, where a darker coloration tends to indicate a higher resistance to pathogenicity (Shigo and Hillis, 1973; Hillis, 1987; Schwarze et al., 2000a). Also, heart rot fungi can grow in, and even invade fresh sapwood where the living cells in response form reactions zones (wall 1–3 of CODIT) to subdue its growth (Shain, 1967, 1971), as in the case of the fungi *Ganoderma* spp, *I. hispidus* (white rot fungi) and *Sparassis crispa* (brown rot fungus) on a range of angiosperm and conifer species (**Figure 3E**). The heart rot fungi with this capability are referred to as specialized opportunists, and are generally weakly pathogenic against well enforced reaction zones (Pearce and Woodward, 1986; Pearce, 1991) (**Figures 3C,D,F**). To clarify here, the use of the term pathogen may seem inappropriate for fungal decay species that are ordinarily confined to the nutrition from dead tissue (i.e., saprotrophs). However, given the right conditions, these species can become pathogenic (weak to moderately), where the integrity of reaction zones can be compromised, although they are usually upheld. Studies only document wood decay fungi that are weakly (saprotrophic) or strongly invasive (facultative pathogenic; Deflorio et al., 2008). For instance, a definite feature of highly invasive wood fungi is their preference to selectively degrade polyphenols (e.g., *Ganoderma adspersum, Phellinus noxius*), whereas saprotrophic fungi such as *Ganoderma lipiense* (Supplementary Table S1) and most brown rot fungi do not breakdown the latter substances. Also, these fungal organisms are very important in the recycling of nutrients that would otherwise be locked into the heartwood of long-lived trees.

Unspecialized opportunists can also invade dysfunctional sapwood, where O_2_ levels are higher and where many of the RAP cells are already dead upon the formation of previous reaction zones. This takes place usually after wound occurrence or where previous infections have weakened sapwood response, e.g., from the white rot *Chondrostereum purpureum* (Rayner and Boddy, 1988). All other fungal strategies occur in the sapwood where the living cells can actively respond. Fungi that use active pathogenesis as a strategy (e.g., *Armillaria mellea, Heterobasidion annosum, Stereum hirsutum*) are aggressive and can penetrate wood directly or through small wounds, often via the roots, using specialized enzymes that can readily attack and kill RAP for their nutrient supply before continuing to colonize more sapwood tissue. Contrary to heart rot fungi, most fungi that use the active pathogenesis mechanism spread in a centripetal direction, starting at the inner bark, where they kill cambial cells thus establishing sufficient inoculation, before moving inward toward the functional sapwood.

Canker rot pathogens of the Basidiomycota (e.g., *I. obliquus* on *Betula* spp.) tend to utilize canker formation (a wedge shaped zone of necrotic tissue) as a way to access sapwood, where once established, the barrier zone of wall 4 of CODIT fails to function effectively owing to repeated fungal attacks of the bark from the sapwood side. The attacks keep the access open to the xylem tissue for further reinvasions, eventually girdling the tree outright (True et al., 1955; Shigo, 1969, 1984; Blanchette, 1982a,b; Biggs, 1992).

Another ability of both brown and white rot is the secretion of oxalic acid, which can lower the optimum pH of surrounding wood tissue, enabling fungal enzymes to challenge xylem defenses by penetrating cell walls of RAP with greater ease (Dutton and Evans, 1996; Nagy et al., 2012). Calcium oxalate crystals, often associated with protection against herbivory and found in secondary phloem of conifers (Hudgins et al., 2003) have also been observed in the RP of the sapwood, and in the dead RP of heartwood in a number of angiosperm species at an advanced stage of decay (von Aufsess, 1974; Muhammad and Micko, 1984). Muhammad and Micko (1984) showed a strong relationship between the accumulations of calcium oxalate crystals in the parenchyma vacuoles and decay by the white rot fungus, *Fomes fomentarius*. Why this relationship occurs is uncertain, but the conversion of calcium oxalate ions into crystals is presumably to the benefit of the host. Much work remains to understand this aspect of fungi-plant dynamics. An example of calcium oxalate crystals in the RP of *Ziziphus obtusifolia* is shown in **Figure 1**, where, in this case, they may be acting in defense as part of wall 3, or simply localizing toxic compounds.

## RAP and Tree Longevity

The inherent anatomical constitution of a tree and its investment in defense components are both major factors in determining how fast a tree grows and how long it lives. Generally, investment in defense (encompassing wood density) is counterproductive to fast growth, so trees that do invest tend to grow slowly and live longer, while the opposite tends to be true for species that do not invest (Coley et al., 1985; Loehle, 1988; Lamarre et al., 2012; Herms and Mattson, 1992). Angiosperm pioneer species are a good example for the latter strategy, where to be successful they must grow fast but at the expense of defense investment. However, during rapid growth, fast growing species can keep pace with decay advancement and maintain the capacity to close wounds rapidly (Coley et al., 1985). While deciding on traits that promote longevity in angiosperms is more complex, longevity in conifers was found to be strongly correlated with defense against decay-causing fungi (Loehle, 1988). ‘Safe’ narrow tracheids and a torus-margo bordered pit mechanism act to localize embolism and decay, a different functional approach compared to the angiosperms, which could be a partial explanation for conifers having fewer RAP cells and in particular AP cells.

The best predictor of longevity in angiosperms was found to be volumetric heat content (J cm ^-3^ K^-1^) defined as the level of investment into structural (e.g., bark thickness) and chemical defense (e.g., polyphenolic compounds and latex; Loehle, 1988). Hence, how RAP fractions and spatial arrangements play a role in tree longevity is unclear. Wood containing more RP tends to be denser, while wood with more AP tends to be less dense, and these opposite trends result in an overall relationship between total RAP and wood density that is fairly weak (Taylor, 1969; Martínez-Cabrera et al., 2009; Poorter et al., 2010; Zheng and Martínez-Cabrera, 2013). Some tropical fast growing and short-lived pioneers of the tropics such as *Cecropia* sp., *Ceiba pentandra* and *Ochroma pyramidale* have very high RAP fractions, with the latter having RAP upward of 90% (Wagenführ, 2007). Temperate pioneer species that exhibit fast growth tend to have comparatively lower RAP fractions. For example, *Populus* sp. and *Liriodendron tulipifera* have 12.3 and 19% RAP, respectively (Wagenführ, 2007), with AP fractions of both being absent or rare. Not surprisingly, *Populus* spp. had among the lowest heat content, where, in addition, species of this genus are generally considered poor compartmentalizers (wall 1–3), although much variation in the degree of compartmentalization exists even within the same species (Shigo et al., 1977). High RAP fractions in tropical pioneer species is clearly not correlated with longevity; however, RAP involvement in hydraulic maintenance may serve as a trade-off between growth rate and defense, where rapid secondary growth allows the tree to keep pace with the advancing decay. Heart rot is another important consideration of long-lived tree species, which results in a hollowed core to the interior of the barrier zone (wall 4 of CODIT; Shigo, 1984). Providing stem breakage does not occur due to an insufficient amount of remaining wood, species that are prone to heart rot, but have a highly effective barrier zone, may prevent any further decay spread in a centrifugal direction, thus prolonging tree life. This area of research merits further investigation.

## Conclusion

The importance of the role of RAP in defense against pathogens is often overlooked and, in general, has been viewed in past studies as not playing as significant a role in defense when compared to the non-living components of the sapwood and the otherwise physical structure of the xylem at the time of decay (Hillis, 1977; Boddy and Rayner, 1983). Although the non-living part of the xylem is important, and forms a critical component of the CODIT model, the role of living cells is equally vital, considering the dynamic continuum that exists between symplastic and apoplastic tissues of the sapwood through to the non-living heartwood, where dead parenchyma also plays an important role in the latter. Studies carried out into the many functions of RAP have clearly demonstrated this continuum, e.g., the relationship between contact cells and vessels, where tyloses form to halt decay or pathogen spread. There clearly appear to be trade-offs between RAP functions, where RAP proportions might not serve to be as strong an indicator of defense capabilities as their spatial arrangement. For instance, paratracheal AP arrangements may play a greater role in hydraulic maintenance (**Figure 2E**), while banded AP running between rays act in unison with the growth increments (when present) to halt decay radially (wall 2 of CODIT). We must also consider the trade-offs that exist between RP and AP, where more work is required to determine the defensive roles of these two living tissues of different cambial derivation. An important approach would be to look more closely at structure-function relationships in wood, especially RAP spatial arrangements and amounts, in order to form a stronger base for developing and testing new hypotheses linked with CODIT. In addition, three-dimensional reconstructions using high-resolution computed tomography (Brodersen, 2013; Brodersen and Roddy, 2016), as we have illustrated, will help pave the way for a greater understanding of the interconnectivity that exists between symplastic and apoplastic networks, and ultimately the CODIT concept. More integration of the CODIT model with related models that are focused on plant hydraulics, such as sectoriality and hydraulic redundancy, might contribute to understanding interconnectivity from the whole plant down to the organ and cell level. This point is especially important when maintaining hydraulic function, which is likely to play a key indirect role in keeping fungal pathogens from gaining access and spreading throughout the sapwood. Hence, hydraulic models could complement the CODIT model.

On a broad scale, trade-offs in defense against fungal pathogens may vary between climates (e.g., temperate and tropical) and between wet and dry zones, with these variables being closely related to phenology (i.e., temperate deciduousness) and growth form (e.g., pachycauls of dry subtropical regions). Future avenues of research should focus on the trade-offs that may exist between species of different climate zones, rainfall gradients within climate zones, and growth forms (e.g., lianas, shrubs, and pachycauls), while also taking appropriate care to reduce any phylogenetic noise. An investigation into trade-offs between RAP at the family level with representatives across a range of biomes would be a useful start, for example in the Malvaceae. Simple wounding experiments could test to see if other malvaceous woody plants have parenchyma in the secondary xylem that can dedifferentiate, such as that of *Adansonia* spp. (Fisher, 1981). Also, trade-offs between bark defense and reaction zones in sapwood should be further tested in a range of tropical and temperate species, including woody plants form dry and wet regions. Regions where herbivores or fire pose a greater threat than pathogens might result in selection for thicker barks that respond faster to wounds at the expense of strong reaction zones. Also, more carefully planned experiments on a range of species from different climatic zones might further support the hypothesis that wall 4 of CODIT is stronger in species with weak reaction zones, supporting previous studies that hydraulic upkeep overrides physical and chemical barriers as a means to restrict fungal growth (Boddy and Rayner, 1983; Rayner and Boddy, 1988; Baum and Schwarze, 2002). Finally, at a cellular level, we should focus on the role of contact parenchyma of both RP and AP and the possible relationship between the amorphous layer, pectin, gel, and tylosis formation. More research is clearly required to further elucidate the contributions of RAP to tree defense mechanisms, and the overall framework of disease resistance in trees, including genetic variation, environmental influences and microbiome interactions.

## Author Contributions

HM and SJ provided the CODIT illustration (**Figure 2**), and coordinated the writing. CB provided the MicroCT images (**Figure 1**), went through the text and made useful suggestions. FS provided the image plate to accompany the text (**Figure 3**), helped with the development of the Supplementary Table S1, and made useful comments, while providing additional ideas for the main text.

## Conflict of Interest Statement

The authors declare that the research was conducted in the absence of any commercial or financial relationships that could be construed as a potential conflict of interest.

## Abbreviations

AP: axial parenchyma
CODIT: compartmentalization of decay in trees
NSC: non-structural carbohydrate
RAP: radial and axial parenchyma
RP: ray parenchyma
