# Supplementary material for: The Parenchyma of Secondary Xylem and Its Critical Role in Tree Defense against Fungal Decay in Relation to the CODIT Model
Source: Front Plant Sci. 2016 Nov 9;7:1665. doi: 10.3389/fpls.2016.01665 (PMC5101214; doi:10.3389/fpls.2016.01665)
Supplement: Supplementary file 1 [file Table_1.DOCX]

**Table S1. The role of RAP in defense against pathogenic fungi in a range of northern hemisphere temperate tree species, and source(s) of the literature.**

| Decay type | Fungal Species | Host species | Effect on RAP | References |
| --- | --- | --- | --- | --- |
| Brown rot  (Basidiomycota) | *Fistulina hepatica*  Most commonly on oaks and sweet chestnuts. Oak timber that has been infected with this parasite is greatly valued by wood turners and cabinet makers, who sometimes refer to it as 'brown oak' Can switch to a soft rot mode. | *Quercus robur* | Preferential degradation of polyphenolic deposits in RP and AP. | Schwarze et al., 2000c |
|  | *Laetiporus sulphureus*  Most often on beech, oak, chestnut, sycamore, yew and less frequently cherry and other hardwoods. Heart rot decay strategy where sapwood is seldom infected. | *Quercus robur* | Birefringence exhibited by both RP and AP, where the cell walls remained intact with no structural changes, even after a 12 week incubation period. | Schwarze et al., 2003 |
|  |  | *Robinia pseudoacacia* | At later stage, chlamydospores spread through the ray cells at some distances from decay, which germinate under the right conditions. Otherwise, cell walls of RAP remain intact. | Schwarze et al., 2003 |
|  |  | *Acer pseudoplatanus* | Marginal apotracheal AP showed no degradation and appeared to strongly support the heavily lignified fibers at growth-ring boundary (wall 2 of CODIT). Treatment of sycamore wood with *L. sulphureus* enables detection of annual rings. | Schwarze, 2007; Deflorio et al. 2005 |
|  |  | *Betula pendula* | Apotracheal marginal AP effective as part of wall 2. | Schwarze et al., 2003; Schwarze, 2007 |
|  | *Fomitopsis pinicola*  Rare in Britain and Ireland but common in most countries of mainland Europe, in Scandinavia  *F. pinicola* is very common, and in Slovenia it is quite often seen the trunks of aged birches and Beech trees as well as on conifers. Saprophyte on dead or weakened trees. | *Picea abies* | Xylem ray parenchyma and epithial cells exhibited birefringence even at advanced stages of degradation. | Schwarze et al., 2003; Schwarze, 2007 |
|  |  | *Fagus sylvatica* | Strongly lignified fibre tracheids passively resist spread of decay and exhibit birefringence even at advanced stages of degradation. | Schwarze et al., 2000a |
| White rot  Selective delignification  White rot  Simultaneous white rot | *Perenniporia fraxinea*  [Parasitic](http://www.mushroomexpert.com/glossary.html#parasite) on living ash trees (species of *Fraxinus* and *Robinia pseudoacacia*), especially [white ash](http://www.mushroomexpert.com/trees/fraxinus_americana.html) (*Fraxinus americana*) (also occasionally reported on other hardwoods). | *Robinia pseudoacacia* | RAP cells preferentially degraded during early stages of delignification. | Schwarze, 2008 |
|  | *Heterobasidion annosum*  Most conifer species are susceptible to this decay fungus causing serious decay in the latter’s roots and lower stems. | *Picea abies* | This fungus can effectively breach the reaction zones laid down by RP through the release of oxalic acid, a compound that reduces surrounding pH allowing digestive enzymes to degrade cells more easily, at the heartwood-sapwood boundary. | Shimada et al., 1997; Nagy et al., 2012. |
|  | *Ganaderma pfeifferi*  Commonly occurs on Beech and Oak. Moderately invasive - mostly confined to the heartwood. | *Fagus sylvatica, Quercus robur* | Found to show preferential degradation of the middle lamellae of RP. | Schwarze, 2008; Schwarze and Ferner, 2003 |
|  | *Ganoderma adspersum*  Commonly found on limes, oaks, beech, birch, London plane and Horse Chestnut. Strongly invasive. | *Fagus sylvatica* | Found to preferentially degrade the polyphenolic compounds of AP and tyloses at in early stages of colonization. Caused high weight losses in wood with Reaction Zone. Speculated to be related to an ability of hyphae to respond to chemical stimuli, Cell walls of RP, although inhabited by hyphae, remained intact. | Schwarze and Baum, 2000;  Rayner and Boddy, 1988; Schwarze and Ferner, 2003 |
|  | *Ganoderma lipsiense*  Commonly found on beech, birch, poplar, horse chestnut. Weakly invasive – saprophyte mostly confined to the heartwood. | *Platanus x hispanica* | Failed to degrade the polyphenolic compounds of AP and RP or tyloses in reaction zone of London plane (*Platanus x hispanica*). Caused low weight losses in wood with RZ. *G. resinaceum* was shown to have similar degrading mechanisms, but caused moderate weight losses in wood with reaction zones. | Schwarze and Ferner, 2003 |
|  | *Inonotus hispidis*  Commonly found on London plane and Common ash. Mildly invasive. Can switch to a soft rot mode. | *Platanus x hispanica* | Strong wall 3 (RP) compared to *Fraxinus excelsior*. However, hyphae can breach reactions zones of RP via a soft rot mode, where the fungus avoids the polyphenol induced lumina by channelling through cell walls. Even at advanced stages, intercellular space of RP remain intact due to the copius production of polyphenols within intercellular spaces and cell lumina of RP. AP was not effected by cavity formation (soft rot), but pits in cells walls had enlarged. Also, thinning of cell walls was apparent in AP, under 12 week incubation period, but not in RP. | Schwarze et al., 1995  Schwarze and Fink, 1997 |
|  |  | *Fraxinus excelsior* | Early degradation in cell walls of RP was found, which was in contrast to *P. x hispanica*. Thought to be due to a low moisture or variation in lignin distribution. | Schwarze et al., 1995 |
|  | *Trametes versicolor*  Grows mainly on dead hardwood, including stumps and standing dead trees as well as fallen branches. | *Fagus orientalis* | Was found to completely destroy RAP in this species, where the destruction of RP paved the way for further advancement of sapwood tissue. | Bari et al., 2015 |
|  |  | *Liquidambar styraciflua, L. orientalis* | Fiber cell walls degraded preferentially, with vessels and RP degraded later at an advanced decay stage. RP were shown to be very resistant to decay of this fungal species. Considered a selective white rot by Yilgor et al. (2013). | Obst et al., 1994  Yilgor et al., 2013 |
|  | *Armillaria mellea*  Primary parasite on a range of deciduous trees or young susceptible conifers. Can switch to a soft rot mode. | *Acer pseudoplatanus* | Hyphae observed in the cells lumina of RP; however, the living fibers, along with marginal AP concentrated at the annual-ring boundary. This fungus has the ability to degrade all cell wall constituents, though tends to prefer walls of low lignin concentration at the earlier stages of decay. | Schwarze et al., 2000b; Schwarze, 2007. |
|  | *Meripilus giganteus*  Common throughout most of Britain and Ireland, the fungus is scarcer in northern Scotland. This species occurs in most of mainland Europe.  Mildly invasive - can switch to a soft rot mode. | *Tilia platyphyllos* | Hyphae abundant in lumina of vessels, RP and AP. Marginal apotracheal AP, reticulate AP, and RP were preferentially degraded. Progressive delignification occurs in the secondary cell wall of the RP, where delignification of middle lamellae caused the separation of cells walls. Advanced decay leads to complete degradation of RP, prior to delignification of fiber secondary walls. | Schwarze and Fink, 1998. |
|  | *Pleurotus ostreatus*  Commonly on hardwoods e.g. alder, ash, beech, birch, chestnut, elm, horse chestnut, Occurs in both heart- and sapwood, causing a white, flaky rot*.* | *Fagus orientalis* | Total destruction of RP. Ray cytoplasm degraded prior to cell wall, before entering cell lumina. Complete delignification thought to be attributable to low nitrogen content of wood. | Bari et al., 2015 |
|  | *Fomes fomentarius*  Commonly found on beech and birch. Latent coloniser or endophyte activated via dormant chlamydospores. | *Fagus sylvatica* | RP and AP were affected only after 16 weeks of the incubation period, showing that over other cell types, they show more resilience compared to fibers. | Baum et al., 2003 |
| Soft rot | *Kretzschmaria deusta*  Found in most temperate regions of the Northern Hemisphere, particularly where lime, beech and oaks grow. Moderately invasive on beech, strongly invasive on lime.  Strong reaction zones recorded from walls 1-3 of CODIT. | *Fagus sylvatica*  *Tilia platyphyllos* | Both RP and AP were found to be heavily occluded with polyphenols in reaction zones. Deposits were slowly degraded.  Both RP and AP were found to be weakly occluded with polyphenols. In RP the polyphenols were mostly deposited in the pit apertures. All deposits were rapidly degraded. | Schwarze and Baum, 2000.  Baum and Schwarze, 2002 |

Bari, E., Nazarnezhad, N., Kazemi, S.M., Ghanbary, M.A.T., Mohebby, B., Schmidt, O., et al. (2015). Comparison between degradation capabilities of the white rot fungi *Pleurotus ostreatus* and *Trametes versicolor* in beech wood. *Int*. *Biodeterior*. *Biodegradation* 104, 231-237. doi: 10.1016/j.ibiod.2015.03.033

Baum, S., Schwarze, F.W.M.R. (2002). Large-leaved lime (*Tilia platyphyllos*) has a low ability to compartmentalize decay fungi via reaction zone formation. *New Phytol.* 154, 481-490. 10.1046/j.1469-8137.2002.00390.x

Baum, S., Sieber, T.N., Schwarze, F.W.M.R., Fink, S. (2003). Latent infections of *Fomes fomentarius* in the xylem of European beech (*Fagus sylvatica*). Mycol. Prog. 2, 141-148. doi: 10.1007/s11557-006-0052-5

Deflorio, G., Hein, S., Fink, S., Spiecker, H., Schwarze, F.W.M.R. (2005). The application of wood decay fungi to enhance annual ring detection in three diffuse-porous hardwoods. *Dendrochronologia* 22, 123-130. doi:10.1016/j.dendro.2005.02.002

Nagy, N.E., Kvaalen, H., Fongen, M., Fossdal, C.G., Clarke, N., Solheim, H., et al. (2012). The pathogenic white-rot fungus *Heterobasidion parviporum* responds to spruce xylem defense by enhanced production of oxalic acid. *Mol. Plant Microbe Interact.* 25, 1450-1458. doi: 10.1094/MPMI-02-12-0029-R

Obst, J. R., Highley, T. L., and Miller, R. B. (1994). “Influence of lignin type on the decay of woody angiosperms by *Trametes versicolor*,” in, Biodeterioration Research 4, G. C. Liewellyn, et al. (eds.), Plenum Press, New York.

Rayner, A.D.M., Boddy, L. (1988). *Fungal Decomposition of Wood: Its Biology and Ecology*. Chichester: John Wiley International.

Schwarze, F.M.W.R, Baum, S., Fink, S. (2000b). Resistance of fibre regions in wood of *Acer pseudoplatanus* degraded by *Armillaria mellea.* *Mycol. Res.* 104, 126-132. doi: 10.1017/S0953756200002525

Schwarze, F.M.W.R, Baum, S., Fink, S. (2000c). Dual modes of degradation by *Fistulina hepatica* in xylem cell walls of *Quercus robur*. *Mycol. Res*. 104, 846-852.

Schwarze, F.M.W.R, Ferner, D. (2003). Ganaderma on trees - differentiation of species and studies of invasiveness. *Arboric. J.* 27, 59-77. doi:10.1080/03071375.2003.9747362

Schwarze, F.W.M.R, Baum, S. (2000). Mechanisms of reaction zone penetration by decay fungi in wood of beech (*Fagus sylvatica*). *New Phytol*. 146, 129-140.

Schwarze, F.W.M.R. (2007). Wood decay under the microscope. *Fungal Biol. Rev.* 21, 133-170. doi:10.1016/j.fbr.2007.09.001

Schwarze, F.W.M.R. (2008). *Diagnosis and prognosis of the development of wood decay in urban trees*. Australia: ENSPEC Pty LtD.

Schwarze, F.W.M.R., Fink, S. (1997). Reaction zone penetration and prolonged persistence of xylem rays in London plane wood degraded by the Basidiomycete *Inonotus hispidus*. *Mycol. Res.* 101, 1207-1214. doi: 10.1017/S0953756297003808

Schwarze, F.W.M.R., Fink, S., Deflorio, G. (2003). Resistance of parenchyma cells in wood degraded by brown rot fungi. *Mycol. Prog.* 2, 26-74. doi: 10.1007/s11557-006-0064-1

Schwarze, F.W.M.R., Lonsdale, D., Fink, S. (1995). Soft-rot and multiple T-branching by the basidiomycete *Inonotus hispidus* in ash and London plane. *Mycol. Res.* 99, 813-820. doi: 10.1016/S0953-7562(09)80732-6

Schwarze, F.W.M.R., Mattheck, C., Engels, J. (2000a). Fungal strategies of wood decay in trees. Heidelberg, Germany: Springer.

Shimada, M., Akamatsu, Y., Tokimatsu, T., Mii, K., Hattori, T. (1997). Possible biochemical roles of oxalic acid as a low molecular weight compound involved in brown-rot and white-rot wood decays. *J. Biotechnol.* 53, 103-113. doi:10.1016/S0168-1656(97)01679-9

Yilgor, N., Dogu, D., Moore, R., Terzi, E., Kartal, S.M. (2013). Evaluation of Fungal Deterioration in *Liquidambar orientalis* Mill. Heartwood by FT-IR and Light Microscopy. *BioRes*. 8, 2805-2826. doi: 10.15376/biores.8.2.2805-2826
